# Exploring hematological alterations and genetics linked to SNV *rs10974944* in myeloproliferative neoplasms among Amazon patients

**DOI:** 10.1038/s41598-024-60090-x

**Published:** 2024-04-24

**Authors:** Jhemerson F. Paes, Dania G. Torres, Deborah C. Aquino, Emanuela V. B. Alves, Erycka A. Mesquita, Miliane A. Sousa, Nelson Abrahim Fraiji, Leny N. M. Passos, Rosângela S. Abreu, George A. V. Silva, Andréa M. Tarragô, Lucivana P. de Souza Mourão

**Affiliations:** 1 Programa de Pós-Graduação em Ciências Aplicadas à Hematologia, Universidade do Estado do Amazonas (UEA), Manaus, AM 69850-000 Brazil; 2 Fundação Hospitalar de Hematologia e Hemoterapia do Amazonas (FHEMOAM), Manaus, AM 69050-002 Brazil; 3 Escola Superior em Ciências da Saúde (ESA/UEA), Av. Carvalho Leal, 1777 - Cachoeirinha, Manaus, AM 69065-001 Brazil

**Keywords:** Myeloproliferative disease, Genetics

## Abstract

BCR::ABL1-negative myeloproliferative neoplasms are hematopoietic disorders characterized by panmyelosis. *JAK2 V617F* is a frequent variant in these diseases and often occurs in the 46/1 haplotype. The G allele of *rs10974944* has been shown to be associated with this variant, specifically its acquisition, correlations with familial cases, and laboratory alterations. This study evaluated the association between the 46/1 haplotype and *JAK2 V617F* in patients with myeloproliferative neoplasms in a population from the Brazilian Amazon. Clinical, laboratory and molecular sequencing analyses were considered. Carriers of the G allele of *rs10974944* with polycythemia vera showed an increase in mean corpuscular volume and mean corpuscular hemoglobin, while in those with essential thrombocythemia, there was an elevation in red blood cells, hematocrit, and hemoglobin. Associations were observed between *rs10974944* and the *JAK2 V617F*, in which the G allele (OR 3.4; *p* < 0.0001) and GG genotype (OR 4.9; *p* = 0.0016) were associated with *JAK2 V617F* + and an increase in variant allele frequency (GG: OR 15.8; *p* =  < 0.0001; G: OR 6.0; *p* = 0.0002). These results suggest an association between *rs10974944* (G) and a status for *JAK2 V617F*, *JAK2 V617F* + _VAF ≥ 50%, and laboratory alterations in the erythroid lineage.

## Introduction

Myeloproliferative neoplasms (MPNs) are clonal diseases characterized by hyperplasia of the myeloid lineage with effective maturation, which results in leukocytosis in peripheral blood, increased erythrocyte mass and possible progression to medullary fibrosis or leukemic transformation^1^. They have an incidence rate of 6 cases per 100,000 individuals and mostly affect white males between 60 and 70 years of age^2^. Polycythemia vera (PV), essential thrombocythemia (ET), and primary myelofibrosis (MF) are the most common *BCR::ABL1-*negative MPNs, though differ in signs, symptoms, hematological and clinical alterations, and genetic findings^3^.

*JAK2 V617F* (dbSNP ID: *rs77375493*) is the main genetic finding in MPNs and has a frequency of 95% in PV cases and between 50–60% in ET and MF cases^4^. This somatic variant triggers the substitution of valine by phenylalanine at codon 617, which alters the pseudo-kinase domain of the JAK2 protein and conditions a constitutive activation of the JAK/STAT signaling pathway^5^.

**Figure 1:**
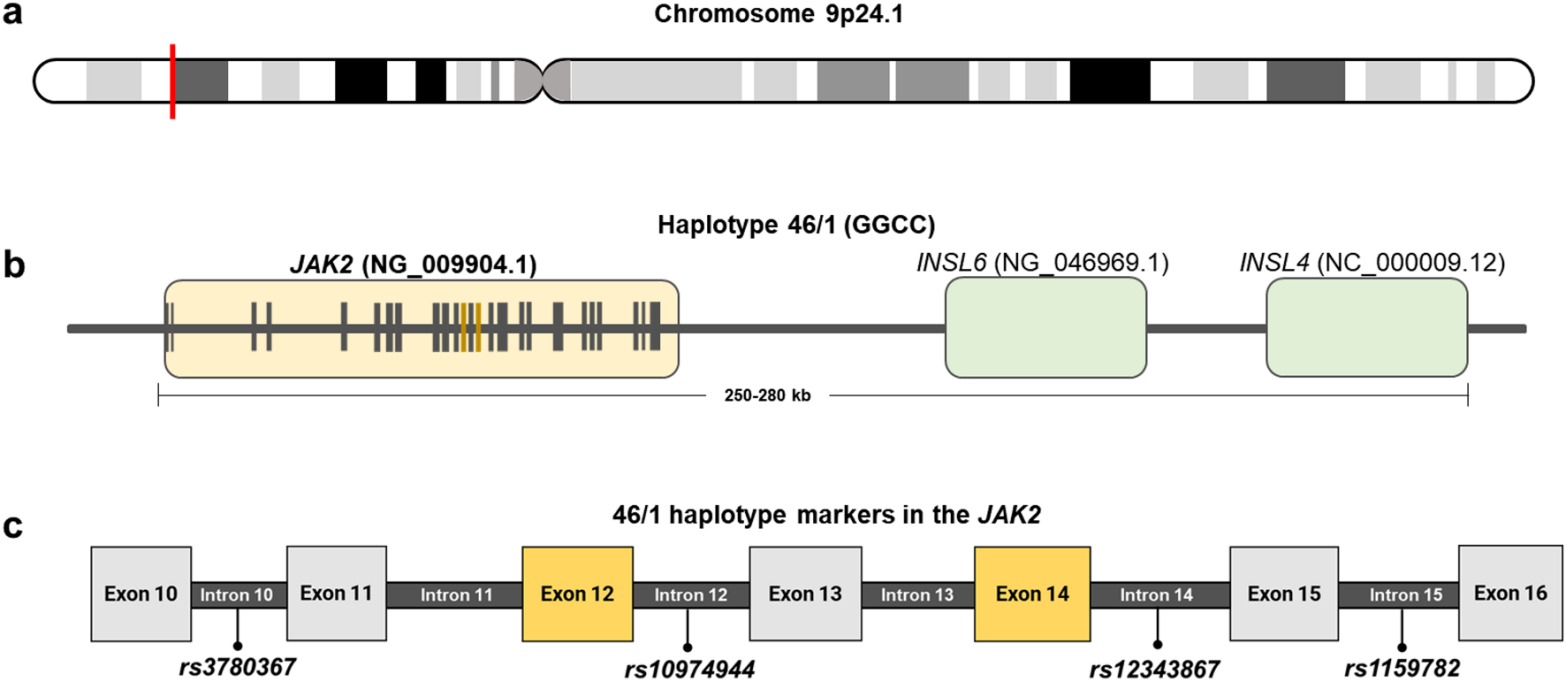
The 46/1 haplotype, (**a**) located on chromosome 9p24.1, (**b**) encompasses the genes *JAK2, INSL6* and *INSL4*. (**c**) Variants in introns 10, 12, 14, and 15 are in strong linkage disequilibrium with the 46/1 haplotype and serve as markers for the detection of this haplotype.

Studies have shown a significant correlation between *JAK2 V617F* and the 46/1 haplotype, a set of germline genetic variations distributed along chromosome 9p.24.1. This haplotype covers regions with a high number of genetic variants in *JAK2* (exons 12 and 14) and is in linkage disequilibrium with the variant *rs10974944* (C > G), located in intron 12 of the same gene^6^ (Fig. 1). Studies indicate that this genetic alteration is a factor that favors the acquisition of *JAK2 V617F* by increasing the mutational rate of JAK2, which can lead to DNA damage and replication errors^7–9^. In addition to being identified in MPN patients of various populations, this haplotype has also been associated with more pronounced alterations in laboratory exams, presence of splenomegaly, inflammatory dysregulation, familial cases of MPNs (increasing the risk of developing any myeloproliferative neoplasm by 5 to 7 times) and abnormal methylation of the gene promoter^10–13^. Therefore, the JAK2 46/1 haplotype confers predisposition to the development of myeloproliferative neoplasms associated with the *JAK2 V617F* mutation (OR = 3.7; 95% CI = 3.1–4.3) and provides a conceptual framework in which a constitutional genetic component is associated with a substantial increase in the risk of acquiring a specific somatic mutation^14^.

In this study, we performed genetic sequencing of intron 12 of the *JAK2* gene to identify the *rs10974944* variant (C > G), in strong linkage disequilibrium with the 46/1 haplotype, in 100 patients with *BCR::ABL1*-negative myeloproliferative neoplasms (polycythemia vera: *n* = 39; essential thrombocythemia: *n* = 61) for whom clinical and laboratory information was available for clinical and laboratory characterization.

## Results

### Characterization of the study population

The study included individuals clinically diagnosed with polycythemia vera (PV) (*n* = 39) or essential thrombocythemia (ET) (*n* = 61), whose clinical-laboratory characteristics are presented in the supplementary material. The female gender was more prevalent among individuals diagnosed with ET (*n* = 48, *p* = 0.002). The median age of the participants ranged between the fifth and sixth decades of life (*p* = 0.441).

Regarding hematological results, the medians of overall red blood cell count (RBC), hematocrit (Ht), hemoglobin (Hb), and total white blood cell count (WBC) were significantly higher in the PV group compared to the ET group (*p* < 0.05) (see Table SI). Other hematological markers, such as mean corpuscular volume (103.9 pg, *p* < 0.0001), mean corpuscular hemoglobin (33.5 fL, *p* < 0.0001), and overall platelet count (467,000 × cells/mm^3^, *p* < 0.0001), were also significantly elevated in the ET group compared to the PV group. Hemorrhagic events were more frequent in patients with ET compared to PV (*p* = 0.003), while the frequency of splenomegaly and thrombotic events did not differ significantly between PV and ET (*p* > 0.05) groups.

**Figure 2:**
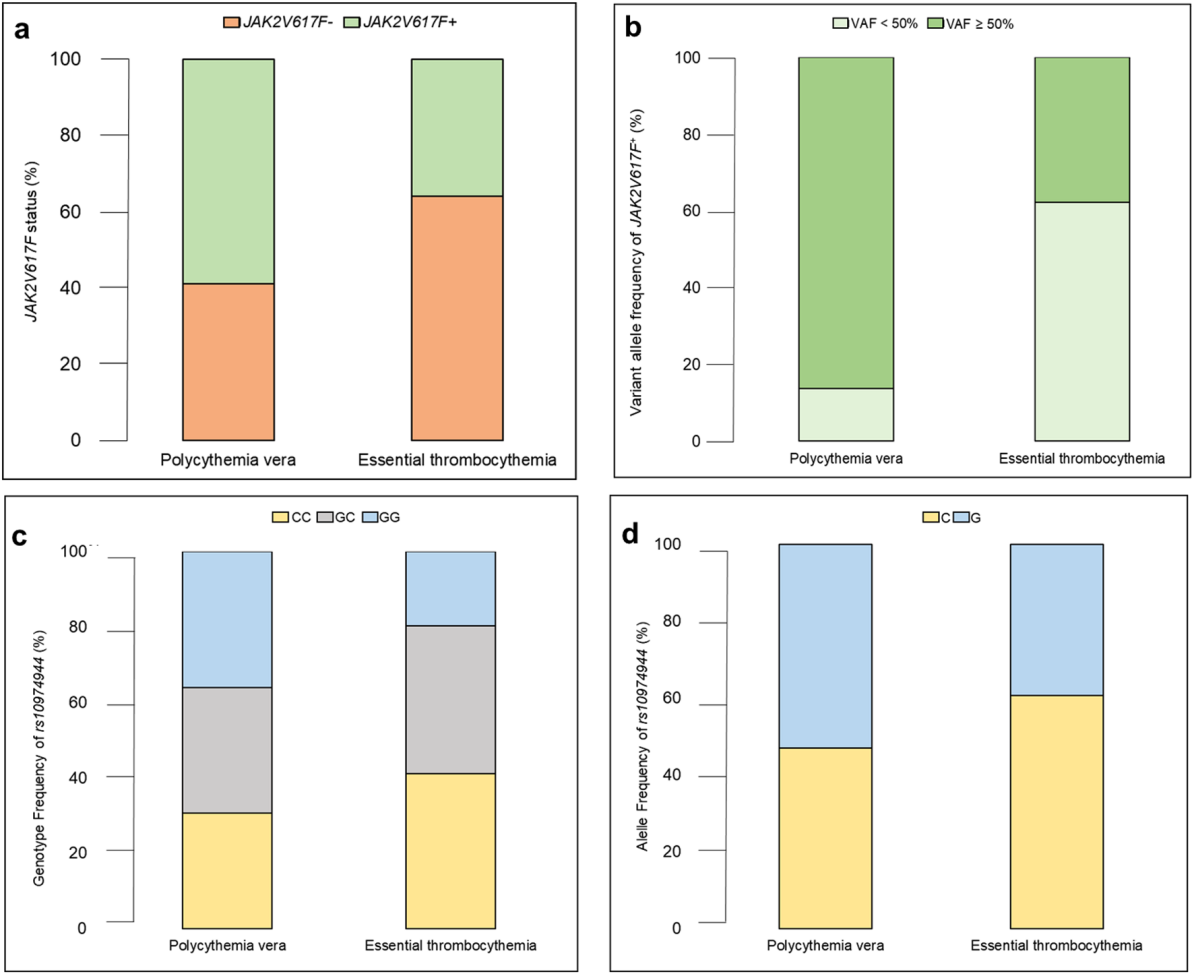
Distribution of genetic data for (**a**) *JAK2 V617F*, (**b**) Variant allele frequency of *JAK2 V617F* + , and (**c**) Genotypic frequency and (**d**) Allelic frequency of *rs10974944* in patients with polycythemia vera or essential thrombocythemia.

Regarding genetic findings, the presence of *JAK2 V617F*^+^ was more frequent in patients with PV (58.9%, *p* = 0.020) (Fig. 2a), and a variant allele frequency (VAF) of ≥ 50% was also more common in patients with this hematologic condition (41%, *p* = 0.005) (Fig. 2b).

A greater frequency of patients with ET (95.1%, *p* < 0.0001) received cytoreductive treatment in comparison to PV patients (66.6%).

### Identified genetic variants

Data on the allelic and genotypic frequency of *rs10974944* (C > G) are presented in Figs. 2c and d. Of all the individuals included in the study, 63% exhibited the *rs10974944* variant (G): 26% in homozygosity (GG) and 37% in heterozygosity (CG). The GG genotype of *rs10974944* was more prevalent in the PV group (36%), whereas CG was more homogeneous between the groups (33.3% in PV and 39.3% in ET). Regarding allelic frequency, the G allele was more frequent in the PV (53.6%) group, and the wild-type allele proved to be more prevalent in the ET (60.7%) group.

Table 1 presents the hematological data of individuals with polycythemia vera and essential thrombocythemia stratified according to the absence or presence of the *rs10974944* (CC and G carriers, respectively). In PV, G carriers showed significantly increased values for MCV and MCH (*p* = 0.030 and *p* = 0.041, respectively), while in ET, patients with the variant exhibited elevated indices of RBC, Ht, and Hb with demonstrated statistical significance (*p* < 0.05).

**Table 1:** Laboratory characteristics of G carriers (*rs10974944*) and individuals without the variant who were diagnosed with polycythemia vera or essential thrombocythemia.

Characteristics, Med, IQR	Polycythemia vera (*n* = 39)			Essential thrombocythemia (*n* = 61)		
	C/C (*n* = 12)	G carriers (*n* = 27)	*p*-value	C/C (*n* = 25)	G carriers (*n* = 36)	*p*-value
RBC (x million/mm^3^)	5.9 [4.7–6.6]	4.8 [4.2–6]	0.188	3.4 [3.1–3.9]	4.1 [3.3–4.7]	**0.029**
Ht (%)	49.8 [43–52.2]	48 [44.1–53.1]	0.866	36.9 [35–39.5]	40.4 [35.9–44.4]	**0.025**
Hb (g/dL)	15.7 [13.1–16.2]	15.4 [13.9–16.6]	0.958	12.1 [11.1–13.0]	13.5 [12–14.6]	**0.004**
MCV (pg)	83.6 [81.7–89.5]	93.7 [86.6–104.9]	**0.030**	106.5 [94.2113.2]	101.5 [90.5–112.6]	0.330
MCH (fL)	27.3 [24.1–29.4]	30.9 [28.1–33.3]	**0.041**	33.6 [31.5–37.5]	32.7 [29.8–36.6]	0.383
MCHC (g/dL)	32.1 [30.4–36.1]	32 [30.6–33.5]	0.816	32.4 [31.6–33.4]	32.5 [32.1–34.2]	0.597
WBC (x cells/mm^3^)	6670 [3835–11,118]	6540 [5190–12,580]	0.911	5260 [3810–7,455]	5410 [4233–6,670]	0.740
Platelets (x per mm^3^)	258,000 [176,250–440,000]	302,000 [0, 0–391]	0.0747	483,000 [368,500–704,500]	440,500 [323,750–1,119,000]	0.127

*RBC* Red blood cell count, *Ht* Hematocrit, *Hb* Hemoglobin, *MCV* Mean corpuscular volume, *MCH* Mean corpuscular hemoglobin, *MCHC* Mean corpuscular hemoglobin concentration, *WBC* White blood cell count. Reference values: RBC: 3.9–5.3 million/mm^3^, Ht: 36–48%, Hb: 12–16 g/dL, MCV: 80–100 fL, MCH: 27–33 pg, MCHC: 32–36 g/dL, WBC: 3,600–11,000 cells/mm^3^, Platelets: 150,000–400,000 per mm^3^.

Significant values are in bold.

Data on the correlation between the G allele and clinical characteristics are shown in Table 2. A significant correlation was observed between the G allele and thrombotic events in patients with PV (*p* = 0.041) and a similar trend in ET, however, without significance statistics (*p* = 0.073). These data suggest that the G allele of *rs10974944* may be associated with an increased risk of thrombotic events in patients with PV.

**Table 2:** Clinical characteristics of G carriers (*rs10974944*) and individuals without the variant who were diagnosed with polycythemia vera or essential thrombocythemia.

Clinical characteristics, *n* (%)	Polycythemia vera (*n* = 39)			Essential thrombocythemia (*n* = 61)		
	C/C (*n* = 12)	G carriers (*n* = 27)	*p*-value	C/C (*n* = 25)	G carriers (*n* = 36)	*p*-value
Splenomegaly						
Yes	2 (16.7)	7 (25.9)	0.692	4 (16)	6 (16.6)	> 0.999
No	10 (83.3)	20 (74.1)		21 (84)	30 (83.3)	
Thrombotic events						
Yes	0 (0.0)	8 (29.6)	**0.041**	3 (12)	12 (58.3)	0.073
No	12 (100)	19 (70.3)		22 (88)	24 (66.6)	
Hemorrhagic events						
Yes	1 (9)	0 (0)	0.307	5 (20)	9 (25)	0.762
No	11 (91)	27 (100)		20 (80)	27 (75)	

Significant values are in bold.

### Distribution of variants in patients stratified according to *JAK2 V617F* status and variant allele frequency

Considering the possible association of *rs10974944* with *JAK2 V617F*, the genotypic frequency analysis of *rs10974944* (C > G) was performed according to the positive (+) or negative (−) status of *JAK2 V617F* and its variant allele frequency (VAF), with data described in Table 3.

**Table 3:** Distribution of single nucleotide variants (SNVs) in MPN patients stratified by *JAK2 V617F* status and variant allele frequency.

Genotype/ allele	*JAK2V617F* status				*JAK2V617F*_VAF				
	*JAK2V617F*^+^(*n* = 45)	*JAK2V617F*- (*n* = 48)	OR (IC 95%)	*p*-valor	≥ 50% (*n* = 20)	< 50% (*n* = 25)	OR (95% IC)	*p*-valor	
rs10974944*; n (%)									
CC	9 (20)	24 (50)	**0.24(0.10**–**0.61)**	0.002	2 (10)	7 (28)	0.3 (0.05–1.6)	0.26	CC vs CG
CG	18 (37.8)	18 (37.5)	1.07 (0.47–2.46)	0.87	3 (15)	14 (56)	**0.14(0.03–0.60)**	**0.0062**	CC vs GG/CG
GG	19 (42.2)	6 (12.5)	**4.9 (1.8–13.9)**	**0.0016**	15 (75)	4 (16)	**15.8 (3.6–68.7)**	** < 0.0001**	CC vs GG
C	35 (38.9)	66 (68.7)	**3.4 (1.9–6.2)**	** < 0.0001**	7 (17.5)	28 (56)	**6 (2.1–14.8)**	**0.0002**	C vs G
G	55 (61.1)	30 (31.2)			33 (82.5)	22 (44)			
rs10119004; n (%)									
AA	27 (60.0)	20 (41.6)	2.1 (0.9–4.8)	**0.077**	16 (80)	11 (44)	5.1 (1.3–19.6)	0.017	AA vs AG
AG	15 (33.3)	21 (43.8)	0.64 (0.3–1.5)	0.302	3 (15)	12 (48)	0.2 (0.04–0.82)	0.027	AA vs CC/AG
GG	3 (6.7)	7 (14.6)	0.4 (0.1–1.7)	0.381	1 (5)	2 (8)	0.6 (0.05–7.2)	> 0.99	AA vs GG
A	69 (76.6)	61 (67.8)	2.1 (1.1–3.9)	**0.025**	35 (87.5)	34 (68)	**3.3 (1.1–10.0)**	0.043	A vs G
G	21 (23.4)	38 (42.2)			5 (12.5)	16 (32)			
rs10815151; n (%)									
CC	33 (73.4)	24 (50)	2.8 (1.2–6.6)	**0.021**	17 (85)	16 (64)	**3.2 (0.7–13.9)**	**0.176**	CC vs CT
CT	7 (15.5)	20 (41.7)	**0.3 (0.1–0.7)**	**0.0056**	2 (10)	5 (20)	**0.4 (0.1–2.6)**	**0.436**	CC vs TT/CT
TT	5 (11.1)	4 (8.3)	**1.5 (0.4–6.0)**	0.734	1 (5)	4 (16)	0.3 (0.03–2.7)	0.362	CC vs TT
C	73 (81)	68 (70.8)	1.8 (0.9–3.5)	0.1019	63 (90)	37 (74)	**5.5 (1.7–18.2)**	**0.0032**	C vs T
T	17 (19)	28 (29.2)			4 (10)	13 (26)			

*VAF* Variant allele frequency. *In linkage disequilibrium with haplotype 46/1.

Significant values are in bold.

Homozygous individuals for *rs10974944* (GG) showed a significantly higher frequency of *JAK2 V617F*^+^ status and a higher likelihood of being positive for this variant when compared to the CC genotype (42.2% vs 12.5%; OR 4.9; 95% CI 1.8–13.9; *p* = 0.00016) (Table 3). We emphasize the correlation of the *rs10974944* G allele with the V617F variant, which demonstrated a 3.4-fold higher probability of being present in *JAK2 V617F*^+^ individuals compared to individuals carrying the C allele (61.1% vs 38.9%; OR 3.4; 95% CI 1.9–6.2; *p* < 0.0001).

Additionally, the analyses revealed that individuals with the GG genotype of *rs10974944* had a 13.1-fold higher probability of having a VAF greater than 50% when compared to individuals with the CC genotype (75% vs 15%; OR 13.1; 95% CI 1.8–72.3; *p* = 0.004). Regarding the allele, carriers of the G allele showed a sixfold higher risk of having a VAF of ≥ 50% compared to the wild-type allele (C) (82.5% vs 17.5%; OR 6.0; 95% CI 2.1–14.8; *p* = 0.0002). These results demonstrate an association between *rs10974944* and the variation in VAF in *JAK2 V617F*.

Analysis of *rs10119004* and *rs10815151* was also performed. The homozygosity for *rs10119004* AA genotype demonstrated a higher prevalence of *JAK2 V617F* + status in comparison to AG and GG genotypes (60.0% vs 33.3% and 6.7%, respectively; OR 2.1; 95% CI 0.9–4.8; *p* = 0.077). Additionally, individuals with the AA genotype exhibited a significantly elevated likelihood of VAF ≥ 50% compared to AG carriers (80% vs 15%; OR 5.1; 95% CI 1.3–19.6; *p* = 0.017). The allele A showed a higher frequency in *JAK2 V617F* + individuals compared to allele G carriers (76.6% vs 23.4%; OR 2.1; 95% CI 1.1–3.9; *p* = 0.025) and carriers of allele A had a significantly elevated likelihood of VAF ≥ 50% compared to allele G carriers (87.5% vs 32%; OR 3.3; 95% CI 1.1–10.0; *p* = 0.043).

Regarding *rs10815151*, the CC genotype displayed a higher frequency of *JAK2 V617F*^+^ status compared to CT and TT genotypes (73.4% vs 15.5% and 11.1%; OR 2.8; 95% CI 1.2–6.6; *p* = 0.021). Moreover, individuals with the CC genotype showed a higher probability of having VAF ≥ 50% compared to those with CT genotype (85% vs 10%; OR 3.2; 95% CI 0.7–13.9; *p* = 0.176). The C allele exhibited a higher prevalence in individuals with *JAK2 V617F*^+^ status compared to T allele carriers (81% vs 19%; OR 1.8; 95% CI 0.9–3.5; *p* = 0.1019), and significantly increased probability of having VAF ≥ 50% compared to those with the T allele (90% vs 74%; OR 5.5; 95% CI 1.7–18.2; *p* = 0.0032).

### Identified haplotypes

**Figure 3:**
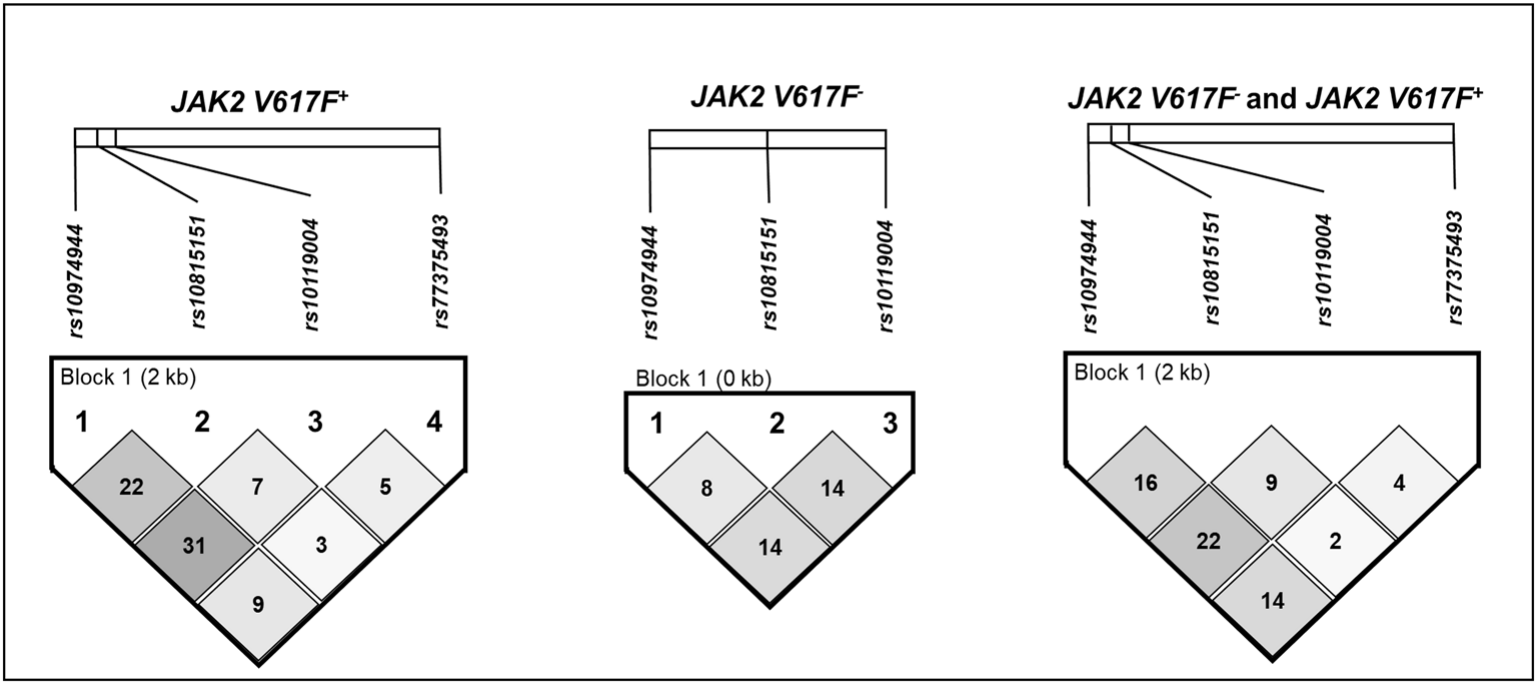
Linkage disequilibrium (LD) structure of *JAK2* intron 12 in patients *JAK2 V617* positive (*JAK2 V617F*^+^) and *JAK2 V617* negative (*JAK2 V617*^*-*^). Numbers in the boxes indicate the value of the LD correlation coefficient (r^2^) multiplied by 100. Lighter shades of boxes indicate a decreased r^2^ value, strong LD is represented by the dark gray box.

The linkage disequilibrium (LD) of *rs10974944* and *JAK2 V617F* (*rs77375493*) is demonstrated in Fig. 3. The variants identified in the analyzed region were included in the haplotype analysis. When these genetic changes are paired, they give rise to nine haplotypes (Table 4). Haplotype analysis revealed that haplotype 2 (*rs10974944G/rs10815151C/rs1011004A/rs77375493T*) was more prevalent in individuals with *JAK2 V617F*^+^ (46.5%; OR 19.6; 95% CI 3.1–208; *p* =  < 0,0001), which indicates a strong correlation between the variants. This information is in accordance with that contained in supplementary figure I and supplementary table III, where it is possible to note the same haplotype 2 more frequent in patients with PV, a neoplasm that presented a higher frequency of the *JAK2 V617F* variant.

**Table 4:** Haplotypes of *JAK2* intron 12 presents in individuals with patients *JAK2 V617* positive (*JAK2 V617F*^+^) and *JAK2 V617* negative (*JAK2 V617*^*-*^).

Haplotype	*rs10974944*	*rs10815151*	*rs10119004*	*rs77375493*	*JAK2 V617F*^+^	*JAK2 V617F *^*-*^	Chi–Square	Odds ratio (95% CI)	*p-value*
1	C	C	G	G	6 (13.9%)	17 (26.4%)	9.025	0.3 (0.1–0.9)	0.0024
**2**	**G**	**C**	**A**	**T**	**20 (46.5%)**	**1 (1.8%)**	**57.79**	**19.6 (3.1–208)**	** < 0,0001**
3	G	C	A	G	5 (11.6%)	14 (26.4%)	5.911	0.3 (0.1–1.1)	0.0015
4	C	T	A	G	4 (9.3%)	15 (28.3%)	10.614	0.2 (0.08–0.8)	0.0011
5	C	C	A	G	0	5 (9.4%)	7.578	0.0 (0.0–0.8)	0.0059
6	C	T	A	T	4 (9.3%)	0	6.434	0.0 (0.08–0.8)	0.0112
7	C	C	G	T	4 (9.3%)	0	7.853	0.0 (0.08–0.8)	0.0051
8	G	T	A	G	0	1 (1.8%)	0.084	0.0 (0.08–11.09)	0.7721
9	G	C	G	G	0	1 (1.8%)	0.316	0.0 (0.08–11.09)	0.5738

Significant values are in bold.

## Discussion

Myeloproliferative neoplasms have characteristic alterations in laboratory exams, as well as genetic findings that permit their identification and differentiation. Findings involving genetic alterations in introns are not yet fully understood, but this scenario is becoming of increasing interest for understanding the etiopathogenic aspects and the role of these DNA regions in these diseases.

Essential thrombocythemia proved to be the most frequent myeloproliferative neoplasm, which are findings that align with the premises established by Torres^15^, who studied a population with *BCR::ABL1*-negative myeloproliferative neoplasms in the state of Amazonas (Brazil). Similar data were described by Macedo^16^, who reported a similar scenario in patients from the states of Paraná and São Paulo who had the same hematologic malignancy, and these data converge with descriptions found in other countries^17,18^.

The age range of individuals was between the fifth and seventh decades of life, which is consistent with what is stated in other studies^19,20^. The progressive accumulation of genetic variations in hematopoietic stem cells and the biological machinery of the DNA repair system^21,22^, an increase or decrease in telomeres^23,24^ and cumulative exposure to risk factors throughout life, such as smoking and obesity^25,26^, may explain the prevalence of this age group in the context of myeloproliferative neoplasms.

Regarding clinical characteristics, polycythemia vera (PV) showed an equal proportion of men and women, while essential thrombocythemia (ET) revealed a majority of cases involving women, and these data are in line with the literature^27,28^. Some studies have demonstrated that women have an increased risk of developing myeloproliferative neoplasms^29^ and a higher likelihood of developing cardiovascular complications and splenomegaly^26^. The reason for this risk is uncertain, but changes in sex chromosomes, hormonal factors and gene expression may be possible contributors to this process^28^. Laboratory data, and thrombotic and hemorrhagic events presented as expected for each neoplasm: PV demonstrated a higher prevalence of increased erythrogram values and ET showed changes in the megakaryocytic series, with a higher risk of hemorrhagic events, as described by the World Health Organization^3^, and in other studies on the subject^27,30^.

Regarding the genetic findings, PV demonstrates a higher prevalence of positive cases for the *JAK2 V617F* variant, since it is directly associated with the specific pathogenesis of this hematologic malignancy^36^ and plays a role in the constitutive activation of the JAK-STAT pathway^5^. It is interesting to note that 58% of our PV population was positive for the variant, which may initially differ from findings commonly described in the literature that point to *JAK2 V617F* frequencies of over 70% in Brazilian, Korean, Chinese, Japanese, and European patients^31–35^.

Our analysis reveals a notable specificity in our population compared to the data documented in the literature, especially in patients with PV, where 42% of these patients did not present the *JAK2 V617F* variant or other pathogenic genetic alterations along the coding region of *JAK2*, as established by WHO diagnostic criteria^3^. This atypical behavior suggests significant gaps in our understanding of the genetic factors underlying the etiopathogenesis of myeloproliferative neoplasms in the Amazonian population. This gap underscores the pressing need for further studies to achieve a more comprehensive understanding of the genetic profile of these diseases and other contributing factors. Therefore, additional studies in our population are recommended, exploring other genes relevant to myelopoiesis and epigenetic regulation, such as *DNMT3A* (*DNA Methyltransferase 3 Alpha*), *NFE2* (*Nuclear factor erythroid 2*), *SF3B1* (*Splicing Factor 3b Subunit 1*), *TET2* (*Tet Methylcytosine Dioxygenase 2*), *ASXL1* (*ASXL Transcriptional Regulator 1*) and EZH2 (*Enhancer Of Zeste 2 Polycomb Repressive Complex 2 Subunit*)^21,52^. Analysis of these genes may provide valuable insights into the genetic behavior of myeloproliferative neoplasms in the Amazonian population and elucidate other factors involved in PV pathophysiology, beyond the known variants in *JAK2 V617F* and *JAK2* exons 12 and 14.

In the literature, the germline haplotype 46/1, identified by the *rs10974944* (C > G) variant, has a well-documented association with *JAK2 V617F*^14,36–38^ as also observed in our study. The data regarding the frequency of the minor allele of *rs10974944* in the Brazilian population and the Amazon region remain scarce, making this study pioneering in this investigation. The absence of previous studies on this variant in the Amazonian population underscores the importance of the current work in filling this gap in the genetic knowledge of this population. However, the frequency of the 46/1 haplotype, associated with *rs10974944*, has been linked to a higher prevalence in patients with myeloproliferative neoplasms, especially those harboring *JAK2 V617F*^+^. This association has been observed not only in other Brazilian populations as described by Macedo et al.^16^ but also in studies conducted across various populations worldwide, including Asian, European, and North American populations, as discussed in one of our previous integrative reviews^13^. Additionally, the ancestral contribution to the Brazilian population, particularly in the Amazon region, is characterized by a mixture of three main ethnic groups: Native Americans (NAM), Europeans (EUR), and Africans (AFR)^39^. Therefore, it is plausible to infer that the genetic behavior of the variant in these populations, as described previously, is similar, thus strengthening the discussion regarding similar behavior in our population.

The high frequency of the G allele of *rs10974944* in individuals positive for *JAK2 V617F* contributes to discussions about the non-random correlation between these two genetic alterations^13,40^ This relationship is in line with another finding from our study, haplotype 2 (*rs10974944*G/*rs10815151C/rs1011004A/rs77375493*T), which strengthens concepts based on the interaction between *rs10974944* (C > G) and *JAK2 V617F* (*rs77375493*—G > T). These propositions are in agreement with findings involving haplotype 46/1 in other Brazilian, Taiwanese, European, Chinese, and Japanese populations^16,32–34,41^, indicating that the possible mechanisms preceding the acquisition of *JAK2 V617F* are not limited to a specific ethnic group; therefore, its evolutionary basis can be considered as a genetic predisposition factor for the disease^8^.

Studies report a higher risk of individuals with the GG genotype of *rs10974944* being positive for *JAK2 V617F*^14,40,42^. Consistent with the results of the aforementioned studies, our population exhibited a four-fold increase in the risk of positive *JAK2 V617F* in individuals with the GG genotype of *rs10974944* (OR 4.1; 95% CI 8–13.9). These findings support the hypothesis of hypermutability, which establishes haplotype 46/1 as a dysregulating agent of the *JAK2* gene, which increases the risk of DNA replication errors and conditions a mutagenic scenario for the acquisition of variants with selective advantages, such as *JAK2 V617F*^43–45^.

The association of *rs10974944* (G) and the *JAK2 V617F* VAF suggests a possible involvement of haplotype 46/1 in clonal expansion. We identified a six-fold higher risk of individuals carrying the G allele of *rs10974944* and *JAK2 V617F* VAF of ≥ 50%. Our data indicate that the marker of haplotype 46/1 may play a role not only in the acquisition of *JAK2 V617F* but is also attributed to clonal expansion, maintenance, and survival. Tefferi^46^ suggests that *JAK2 V617F* is not the initial clonogenic event in MPNs but rather one of several subclones derived from an ancestral clone. This is in accordance with the notes of Pardanani et al.^47^, which support the hypothesis that this haplotype is located in a favorable cis regulatory environment, which facilitates the acquisition of *JAK2 V617F*, and which, in turn, is responsible for clonal expansion and the development of MPNs.

Furthermore, the possible role of acquired uniparental disomy, a genetic event that leads to mitotic recombination associated with neutral loss of heterozygosity of chromosome 9p in MPN patients, reducing both the haplotype and *JAK2 V617F* to a homozygous state^14,48,49^, cannot be ruled out. In this context, cells with both variants theoretically have a selective advantage, which conditions greater myeloproliferative potential and favors the establishment of variant cells over healthy cells, thus explaining the increased VAF in individuals with the combination *rs10974944* (G) + *rs77375493* (T) (*JAK2 V617F*) in homozygosity.

Association between changes in hematological indices, clinical characteristics and the presence of 46/1 is observed in the literature^16,33,50^; however, this is not a consensus among the scientific community^8^. Our data show significant differences in MCV, MCH values in the PV group, and RBC, Hb, and Ht in TE carriers of the G allele of *rs10974944*, which has been observed in previous studies^7,42,51^. The significant demonstration of *rs10974944* with thrombotic events strengthens the use of this variant as a tool for monitoring patients and investigating clinical findings of polycythemia vera. For a more reliable correlation of this correlation, new studies are needed, with more robust populations, to observe the behavior of the variant in relation to clinical and hematological characteristics in PV patients.

The present research is the first to analyze the 46/1 haplotype using the *rs10974944* variant, present in intron 12 of *JAK2*, in a population from the Brazilian Amazon. The results of this study show that the rs10974944 (G) variant has a strong correlation with the *JAK2 V617F*^+^ variant, demonstrated especially in *PV_JAK2 V617F*^+^ patients. A correlation of the variant with a high allelic variant burden of *JAK2 V617F*, thrombotic events and hematological changes was also observed. The variant is a promising possibility for clinical use for investigating and monitoring laboratory changes and/or increased VAF in identified hematological malignancies.

## Materials and methods

### Population

One hundred individuals clinically diagnosed with *BCR::ABL1*-negative myeloproliferative neoplasms were included in the study. The study was conducted from February 2021 to January 2023. Laboratory analysis was performed at the Genomics Laboratory of the Foundation Hospital for Hematology and Hemotherapy of the State of Amazonas.

### Ethical approval

The study was submitted to and approved by the Ethics Committee of the Foundation Hospital for Hematology and Hemotherapy of the State of Amazonas under opinion No. 4,450,813 and certificate of ethical appreciation No. 39991420.6.0000.0009. Written informed consent was obtained from patients. This study complied with Resolution No. 466/2012 of the National Health Council for research involving human subjects and followed the parameters determined by the Declaration of Helsinki.

### Clinical and laboratory data

Clinical data (gender, age, splenomegaly, thrombotic and/or hemorrhagic events) and laboratory data were obtained from medical records.

### Identification of *JAK2 V617F*

The variant was identified according to the parameters and specifications established by Torres et al.^15^ . The negative status for the *JAK2 V617F* variant was confirmed using the allele-specific PCR technique.

### Biological sample and DNA extraction

Venous blood samples were collected in tubes containing EDTA, and DNA was extracted using Brazol (Lgcbio, Brazil), following the manufacturer’s instructions, and stored at − 80 °C.

### Conventional PCR and PCR purification

For the amplification of the DNA region under analysis, a reaction with a final volume of 25 μL was used with 50–100 ng of genomic DNA, Buffer (1x), MgCl_2_ (1.5 mM), forward primer CCAACTGAGTTTCCTTGCAG and reverse primer CTAGGTTAAGAGTATGTGGTTCC (0.4 mM), dNTP mix (0.2 mM), and TAQ (1 U). The PCR products were separated on a 1.5% agarose gel. The PCR product, a 572 bp amplicon, was purified with polyethylene glycol (PEG 8000) (Promega).

### Nucleotide sequencing and sequence analysis

Approximately 5–30 ng of purified PCR product was applied to the sequencing reaction. Nucleotide sequencing was performed using BigDye Terminator v3.1 (Applied Biosystems), following the manufacturer’s recommendations and the primers described above. The products were purified by the EDTA/Ethanol protocol and evaluated in the 3500 XL Genetic Analyzer automatic sequencer (Applied BioSystems, USA), with POP-7 polymer. The sequences were initially analyzed using the Sequencing Analysis software (Applied BioSystems [Thermo Fisher Scientific, São Paulo, Brazil]). Geneious 6.0.6 software (Biomatters, USA) was used to map the variants and obtain contigs for the comparison with the reference sequence *Homo sapiens Janus kinase 2* (JAK2), (NCBI: NG_009904.1).

### Haplotype analysis

Haplotype frequencies were calculated using Haploview software (v.4.2) as a measure of linkage disequilibrium (LD). Haplotypes with frequencies of < 1% were not considered relevant for comparisons. Pairwise degree between nucleotides was analyzed using the LD structure, considering r^2^ values of > 0.8 as strong LD, < 0.8 as weak, and < 0.1 as negative LD. Hardy–Weinberg equilibrium was calculated by comparing estimated and observed genotype frequencies using the χ^2^ test. SNVs with p-values of < 0.001 were considered to be out of Hardy–Weinberg equilibrium.

### Statistical analysis

The obtained results were subjected to the Shapiro–Wilk normality test. Categorical variables were expressed as absolute value (*n*) and relative frequency (%) and were tested using the χ^2^ and Fisher’s exact test with a 95% confidence interval. Numerical variables were expressed as median (Md) and interquartile range [IQR] with 75th percentile through GraphPad Prism v.9.0.2 software. For non-parametric variables, the Kruskal–Wallis test was performed. For both analyses, Dunn’s post-test for multiple comparisons was also conducted using GraphPad Prism v.9.0.2 software. *P*-values of < 0.05 were considered statistically significant.

### Supplementary Information

Supplementary Information.

## Data Availability

The datasets used and/or analyzed during the present study are available from the corresponding author on reasonable request. The GenBank accession number for the nucleotide sequence is PP208825.
