# Supplementary material for: Exploring hematological alterations and genetics linked to SNV rs10974944 in myeloproliferative neoplasms among Amazon patients
Source: Sci Rep. 2024 Apr 24;14:9389. doi: 10.1038/s41598-024-60090-x (PMC11039700; doi:10.1038/s41598-024-60090-x)
Supplement: Supplementary file 1 — Supplementary Information. [file 41598_2024_60090_MOESM1_ESM.docx]

**Supplementary Material**

**Exploring hematological alterations and genetics linked to SNV rs10974944 in myeloproliferative neoplasms among Amazon patients**

Jhemerson F. Paes, Dania G Torres, Deborah C. Aquino, Emanuela Vitória B. Alves^1^, Erycka A. Mesquita, Miliane A. Sousa, Nelson Abrahim Fraiji, Leny Nascimento da Motta Passos, Rosângela Santos de Abreu, George A. V. Silva, Andréa M. Tarragô, Lucivana P. de Souza Mourão

| **Characteristics** | **PV (n=39)** | **ET (n=61)** | **p-value** |
| --- | --- | --- | --- |
| Male/Female, n | 20/19 | 13/48 | 0.024 |
| Age, Med [IQR] | 61 [49-71] | 58 [43.5-72] | 0.441 |
| RBC (x million/mm³), Med [IQR] | 5.57 [4.3-6.1] | 3.75 [3.2-4.5] | <0.0001 |
| Ht (%), Med [IQR] | 48.0 [44.1-52.3] | 37.9 [35.7-42.3] | <0.0001 |
| Hb (g/dL), Med [IQR] | 15.4 [13.8-16.3] | 12.6 [11.6-13.9] | <0.0001 |
| MCV (pg), Med [IQR] | 90.3 [82.6-103.5] | 103.9 [74.9-112.7] | 0.0002 |
| MCH (fL), Med [IQR] | 29.5 [26.7-33.1] | 33.5 [30.3-36.7] | 0.0002 |
| MCHC (g/dL), Med [IQR] | 32 [30.6-33.5] | 32.5 [32-33.6] | 0.156 |
| WBC (x cells/mm³), Med [IQR] | 6,540 [5,150-7,990] | 5730 [3,940-7,110] | 0.009 |
| Platelets (/mm³), Med [IQR] | 294,000 [174,000-391,000] | 467000 [361,500-547,500] | <0.0001 |
| Splenomegaly, n (%) | 9 (23) | 10 (16.3) | 0.441 |
| Thrombotic events, n (%) | 8 (20.5) | 15 (24.5) | >0.999 |
| Hemorrhagic events, n (%) | 1 (2.5) | 14 (22.9) | 0.003 |
| *JAK2 V617F* +, n (%) | 23 (58.9) | 22 (36) | 0.020 |
| VAF *JAK2 V617F* ≥ 50%, n (%) | 16 (41) | 4 (6.5) | 0.005 |
| VAF *JAK2 V617F* < 50%, n (%) | 7 (17.9) | 18 (29.5) |  |
| *JAK2 V617F* not reported, n (%) | 3 (7.6) | 4 (6.4) | >0.999 |

**Supplementary Table I:** Clinical and laboratory characteristics of patients with polycythemia vera or essential thrombocythemia. PV: Polycythemia vera, ET: Essential thrombocythemia, RBC: Red blood cell count, Ht: Hematocrit, Hb: Hemoglobin, MCV: Mean corpuscular volume, MCH: Mean corpuscular hemoglobin, MCHC: mean corpuscular hemoglobin concentration, WBC: White blood cell count, VAF: Variant allele frequency, IQR: Interquartile range, Med: Median. Reference values: RBC: 3.9-5.3 million/mm³, Ht: 36-48%, Hb: 12-16 g/dL, MCV: 80-100 fL, MCH: 27-33 pg, MCHC: 32-36 g/dL, WBC: 3,600-11,000 cells/mm³, Platelets: 150,000-400,000 mm³,

| **Genotype/allele; n (%)** | **PV (n = 39)** | **ET (n = 61)** | **p-value** |  |
| --- | --- | --- | --- | --- |
| CC | 12 (30.7) | 25 (41) | 0.805 | CC vs CG |
| CG | 13 (33.3) | 24 (39.3) | 0.302 | CC vs GG/CG |
| GG | 14 (36) | 12 (19.7) | 0.089 | CC vs GG |
| C | 37 (47.4) | 74 (60.7) | 0.930 | C vs G |
| G | 41 (53.6) | 48 (39.3) |  |  |

**Supplementary Table II:** Allelic and genotypic distribution of rs10974944. PV: Polycythemia vera, ET: Essential thrombocythemia.


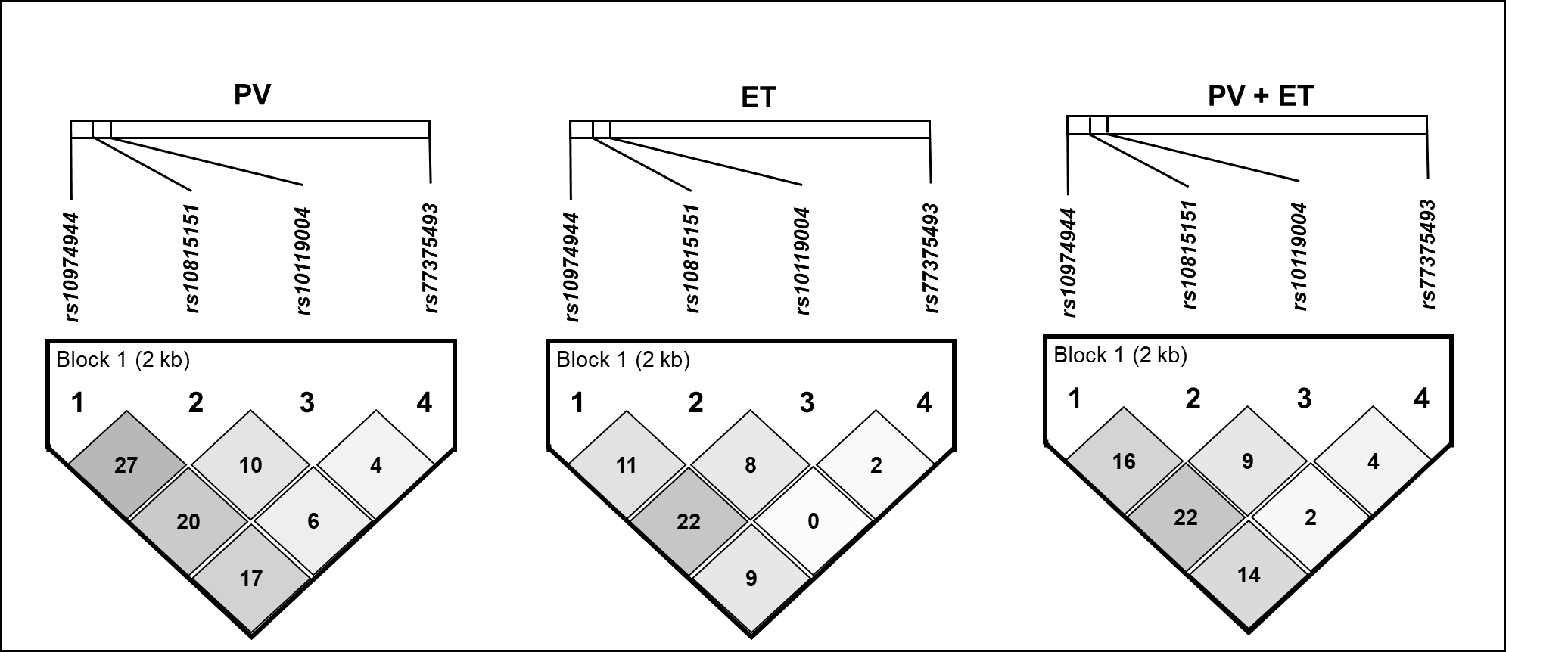


**Supplementary figure I:** Linkage disequilibrium (LD) structure of *JAK2* intron 12 in patients with polycythemia vera (PV) or essential thrombocythemia (ET). Numbers in the boxes indicate the value of the LD correlation coefficient (r^2^) multiplied by 100. Lighter shades of boxes indicate a decreased r^2^ value, strong LD is represented by the dark gray box. A discrete LD is observed between *rs10974944* and *rs1081515*; *rs10974944* and *rs10119004*; and *rs10974944* and *rs77375493* (*JAK2 V617F*).

| **Haplotype** | ***rs10974944*** | ***rs10815151*** | ***rs10119004*** | ***rs77375493*** | **PV** | **ET** | **Chi-Square** | **Odds ratio (CI 95%)** | **p-value** |
| --- | --- | --- | --- | --- | --- | --- | --- | --- | --- |
| 1 | C | C | G | G | 6 (15.3%) | 17 (27.8%) | 3.246 | 0.4 (0.1-1.2) | 0.07 |
| **2** | **G** | **C** | **A** | **T** | **13 (33.3%)** | **8 (13.1%)** | **11.918** | **3.3 (1.2-9.2)** | **0.0006** |
| 3 | G | C | A | G | 6 (15.3%) | 14 (22.9%) | 1.894 | 0.6 (0.2-1.7) | 0.168 |
| 4 | C | T | A | G | 6 (15.3%) | 12 (21.3%) | 0.495 | 0.7 (0.2-2.2) | 0.481 |
| 5 | C | C | A | G | 1 (2.5) | 3 (4.9%) | 1.006 | 0.5 (0.03-3.5) | 0.315 |
| 6 | C | T | A | T | 2 (5.1) | 2 (3.2) | 1.14 | 1.5 (0.2-10.4) | 0.285 |
| 7 | C | C | G | T | 2 (5.1) | 2 (3.2) | 0.28 | 1.5 (0.2-10.4) | 0.597 |
| 8 | C | T | A | G | 1 (2.5) | 1 (1.6) | 0.035 | 1.6 (0.08-31.2) | 0.851 |
| 9 | G | C | G | G | 1 (2.5) | 1 (1.6) | 0.004 | 1.6 (0.08-31.2) | 0.949 |

**Supplementary table III:** Haplotypes of *JAK2* intron 12 present in individuals with polycythemia vera (PV) or essential thrombocythemia (ET).
